# Spinal Involvement of TRPV1 and PI3K/AKT/mTOR Pathway During Chronic Postoperative Pain in Mice

**DOI:** 10.3390/brainsci15010053

**Published:** 2025-01-08

**Authors:** Gabriela Xavier Santos, Tayllon dos Anjos-Garcia, Ana Carolina de Jesus Vieira, Giovane Galdino

**Affiliations:** 1 Center for Experimental Biology, Laboratory of Neuroimmunobiology of Pain, Federal University of Alfenas, Alfenas 37133-840, MG, Brazil; 2 Inapós College, Padre Gervásio National Institute of Higher Education and Postgraduate Studies, Pouso Alegre 37550-121, MG, Brazil; 3 Department of Animal Morphology and Physiology of the Faculty of Agricultural and Veterinary Sciences of São Paulo State University, Jaboticabal 14884-900, SP, Brazil

**Keywords:** chronic postoperative pain, mTOR, PI3K, TRPV1

## Abstract

Background: Chronic postoperative pain (CPOP) is among the main consequences of surgical procedures, directly affecting the quality of life. Although many strategies have been used to treat this symptom, they are often ineffective. Thus, studies investigating CPOP-associated mechanisms may help to develop more effective treatment strategies. Therefore, the present study investigated the spinal participation of the transient potential receptor vanilloid type 1 (TRPV1) and PI3K/AKT/mTOR pathway activation during CPOP. Methods: In this study C57BL/6 male mice were used, and CPOP was induced by muscle retraction and incision. The nociceptive threshold was measured by the von Frey filament test. For pharmacological evaluation, TRPV1 and PI3K/AKT/mTOR inhibitors were administered intrathecally. TRPV1 and PI3K/AKT/mTOR protein levels were evaluated by Western blotting. Results: The results showed that CPOP increased TRPV1 and mTOR protein levels, and pretreatment with the specific inhibitors alleviated CPOP. In addition, pretreatment with the TRPV1 antagonist SB-366791 attenuated mTOR protein levels. Conclusions: The results suggest that TRPV1 and the PI3K/AKT/mTOR pathway are involved in CPOP at the spinal level, and TRPV1 may activate mTOR during this process.

## 1. Introduction

Approximately 300 million people worldwide undergo surgical procedures annually [1]. Although such procedures are needed, they can trigger adverse conditions, including chronic postoperative pain (CPOP). CPOP can affect between 10 and 40% of patients, and this figure is increasing due to the increase in the number of surgeries performed per year [1]. Furthermore, this condition has gained increasing relevance because it causes physical disability, leading to mental symptoms such as anxiety and depression, directly affecting the quality of life [1,2]. In addition, CPOP may have an economic impact on the lives of its sufferers and on the health system, where data in Canada show an expenditure of approximately 40 billion Canadian dollars, both in medical costs and related to lost productivity [3]. Several pharmacological and nonpharmacological strategies have been proposed for CPOP treatment [4]. However, a small proportion of individuals experience a significant reduction in pain [5]. Accordingly, the number of studies seeking to unravel CPOP mechanisms has increased, which can better elucidate its pathophysiology and help in the development of new treatments for its control.

Among the CPOP mechanisms already described, studies carried out in rodents found the participation of glutamate receptors, complement system, purinergic receptors, toll-like receptors, chemokines, and cytokines [1]. Most of these mechanisms have been found at the spinal level, where nociceptive synapses occur. Although these processes have been proposed, additional mechanisms may be involved in pain, of which we highlight the transient receptor potential vanilloid type 1 (TRPV1), a channel permeable to cations [6]. TRPV1 plays an important function in potentiating the transmission of nociceptive impulses in the peripheral nervous system and spinal cord [7].

TRPV1 channels have been demonstrated to be involved in inflammatory and neuropathic pain, expressed in DRG neurons and spinal microglia, respectively. Furthermore, this process involves the activation of some signaling cascades such as protein kinase C (PKC) and Janus kinase (JAK). Once activated, these pathways activate numerous mediators involved with the nociceptive synapse, such as pro-inflammatory cytokines [7]. However, no study has evaluated TRPV1 participation in CPOP induced by the incision and retraction of the muscle model.

In addition to TRPV1, the involvement of the phosphatidylinositol-3-kinase (P13K)/protein kinase B (AKT)/mammalian target of rapamycin (mTOR) pathway has also been demonstrated in different pain models. This pathway has been considered essential for the regulation of translation processes, such as protein synthesis, cell cycle progression, cell survival, and migration [8]. Several studies have also proposed that the PI3K/AKT/mTOR pathway is involved in neuropathic and nociceptive pain [8,9,10]. PI3K/AKT signaling was found to be increased in the spinal cord of rats with neuropathic pain, and this process was associated with facilitated excitatory postsynaptic potential and the phosphorylation of glutamate receptor subunit GluA1. In addition, these effects were reversed by wortmannin, a PI3K blocker [9]. PI3K/Akt/mTOR mRNA expression and protein levels were increased in the spinal cord of rats with neuropathic pain, and this alteration was associated with an increased expression of microglia and astrocytes [8]. In addition to neuropathic pain, a study demonstrated that blocking the Akt/mTOR pathway alleviated pain induced by a plantar incision model in mice and reduced spinal Fos protein expression, a marker of neuronal activity [10].

Once phosphorylated, the PI3K and AKT cascades lead to the activation of the mTOR. This pathway involves intracellular and extracellular signals to control cellular processes, including the expression of genes encoding several inflammatory proteins, such as interleukin 1β (IL-1β), tumor necrosis factor-α (TNF-α), and interleukin 6 (IL-6), which are important players in the nociceptive synapse, as described previously [8,9,10,11].

According to previous studies presented, both TRPV1 and the PI3K/AKT/mTOR pathway share common processes that are involved in nociception, including the production of pro-inflammatory cytokines. Although both TRPV1 and the PI3K/AKT/mTOR pathway are involved in the nociceptive response in different preclinical models of pain, no study has investigated the relationship of both in CPOP genesis. Therefore, unveiling the involvement of this entire pathway may assist in the design of studies for future drug development to control chronic pain. Considering that in vitro evidence shows that once activated, TRPV1 leads to PI3K/AKT/mTOR pathway activation [12], the present study investigated the role of TRPV1 and the PI3K/AKT/mTOR pathway at the spinal level in mice with CPOP.

## 2. Materials and Methods

### 2.1. Animals

Male C57BL/6 mice (20–25 g body weight) were used in this study. The animals were kept in polypropylene boxes with 6 animals in each box. They were maintained in an environment with a controlled photoperiod (12–12 h dark–light cycles), temperature (22–24 °C), and relative air humidity (45–55%). Water and food were provided ad libitum. All animal experimental procedures were approved by the local Animal Care and Use Committee of the Federal University of Alfenas (Brazil) (protocol number 52/2018). The experimental protocol was conducted following the IASP Guidelines [13].

### 2.2. Experimental Design

**Figure 1:**
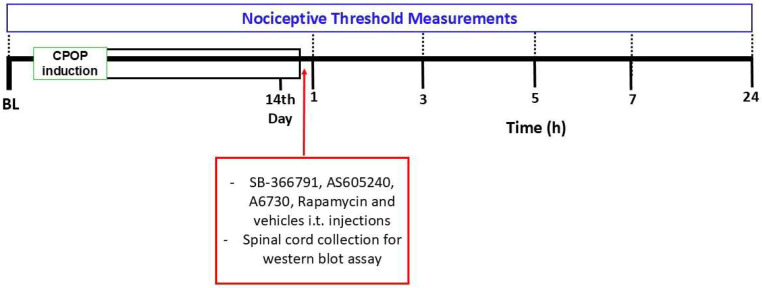
Experimental protocol for behavioral and molecular evaluation of spinal TRPV1/PI3K/AKT/mTOR pathway involvement in chronic postoperative pain (CPOP). BL: baseline. SB-366791: TRPV1 antagonist. AS605240: PI3K inhibitor. A6730: AKT inhibitor. Rapamycin: mTOR inhibitor.

Firstly, the baseline (BL) of the nociceptive threshold was assessed. The nociceptive threshold was also measured on the 14th day of CPOP and after 1, 3, 5, 7, and 24 h of administration of each drug or vehicle (Figure 1). The 14th day was chosen according to a previous study conducted by our group that demonstrated that CPOP was well established in this period [14]. A group of animals received the vehicle (1% DMSO in saline) of each substance to rule out a possible effect of these on the nociceptive threshold. Spinal cord tissue was collected at the time each inhibitor reached its maximum effect.

### 2.3. Postoperative Chronic Pain Model

The chronic postoperative pain (CPOP) model was performed as previously described [13]. Briefly, animals were anesthetized with a solution containing ketamine (80 mg/kg, i.p.) and xylazine (8 mg/kg, i.p.). After trichotomy on the right hind leg, a 1 cm vertical incision was made in the superficial muscle (gracilis) layer of the thigh. Then, the superficial muscles were retracted with a micro retractor revealing the adductor muscles’ fascia [14]. The retraction period lasted 1 h, and the animals were monitored, and additional anesthesia with ketamine and xylazine (at 1/3 of the dose described above) was provided when necessary. After the retraction period, the site was sutured with 4–0 silk suture, and the animals were allowed to recover in their home cages. Instead of a sham group (underwent a false surgery), we used naïve animals, mainly because just an incision in the skin without retraction or a deeper incision could alter the animals’ nociceptive threshold.

### 2.4. Nociceptive Threshold Assessment

The mechanical nociceptive threshold was assessed using the von Frey filament test (Stoeling, Wood Dale, IL, USA). Mice were individually placed in clear glass boxes on a raised metal grid floor to facilitate paw access [15]. Animals were habituated to the apparatus for forty minutes. A mechanical stimulus was applied to the mid-plantar surface using a series of filaments (0.07, 0.16, 0.4, 0.6, and 1.0 g) that was gradually increased until the animal moved its paw away from the stimulus. Stimulation was performed in triplicates and the nociceptive threshold represented the mean values of three consecutive tests [16].

### 2.5. Drugs

The substances used were as follows: TRPV1 antagonist SB-366791 at 2.8 and 28.0 μg/5 µL diluted in 0.9% saline and 1% DMSO [17]. PI3K inhibitor AS605240 at 0.5 and 5.0 μg/5 µL diluted in 0.9% saline and 3% DMSO [18]. AKT inhibitor A6730 at 0.1 and 1.0 µg/5 µL diluted in 0.9% saline and 1% DMSO (1%) [9]. mTOR inhibitor rapamycin at 0.1 and 1.0 µg/5 µL diluted in 0.9% saline [19]. All drugs were purchased from Sigma-Aldrich (Saint Louis, MI, USA).

### 2.6. Intrathecal Injection

Intrathecal injections were made under anesthesia (2% isoflurane in 2 L/min of O_2_). Then, the lumbar region was shaved and cleaned with iodine. The intrathecal injection was performed using a Hamilton syringe and 28-gauge needle (volume 10 µL), first injected at a 70–80° angle and then reduced to 30–45° during drug injection. For injection, the mice were placed in dorsal recumbency to facilitate palpation of the L5–L6 intervertebral spaces [20], and the injected volume of each substance was 5 µL.

### 2.7. TRPV1, PI3K, AKT, and mTOR Protein Level Assessment

Western blotting analysis was performed to evaluate TRPV1, PI3K, AKT, and mTOR protein levels. Thus, animals were euthanized by decapitation 5 h after the injection of each substance or vehicle on the 14th day of CPOP induction. Then, spinal cord samples were removed and homogenized in RIPA buffer containing protease inhibitors (Sigma-Aldrich, Saint Louis, MI, USA). Protein concentration was measured using the Bradford method. Proteins were separated by sodium dodecyl sulfate-polyacrylamide gel electrophoresis (12%) and transferred onto nitrocellulose membranes with a semidry electrophoretic system (Bio-Rad, Hercules, CA, USA). The membrane was blocked for 2 h at room temperature with 5% BSA in PBS, and incubated overnight at 4 °C with mouse anti-TRPV1 (1:5000, Sigma-Aldrich, USA, #A2228), rabbit anti-mTOR (1:1000 Sigma-Aldrich, USA, #SAB5700779), rabbit anti-mTOR phospho Ser 2481 (1:100, Cell Signalling, Danvers, MA, USA, #2974L), rabbit anti-AKT (1:100, Cell Signalling, USA #4691S), rabbit anti-AKT (1:100, Cell Signalling, USA #4691S), rabbit anti-AKT phospho Thr 308 (1:100, Cell Signalling, USA, #13038S), rabbit anti-PIK3 (1:100, Invitrogen, Waltham, MA, USA, #PA5-102022), and rabbit anti-PI3K p110γ (1:1000, Santa Cruz Biotechnology, Dallas, TX, USA, Sc7177). The membranes were incubated with a specific secondary antibody. After that, an enhanced chemiluminescence detection kit (Bio-Rad, USA) was applied for 3 min. Immunoblot images were captured on a chemiluminescence image analyzer (Chemidoc, Bio-Rad, USA), and analyzed using ImageLab software version 2 (Chemidoc, Bio-Rad, USA). The intensity of each band was expressed relative to that of β-actin. In Western blotting analysis, the intensity of each band was expressed relative to that of β-actin for TRPV1 or total protein for PI3K, AKT, and mTOR. Control data were obtained by dividing the data of each control point by the control mean.

### 2.8. Statistical Analysis

**Figure 2:**
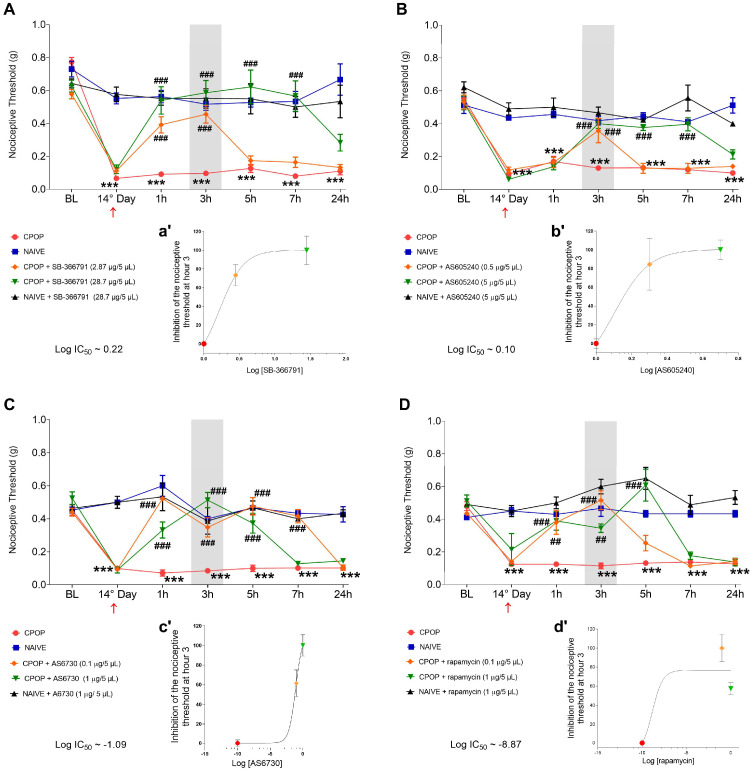
Effect of intrathecal administration of TRPV1 antagonist SB-366791 (**A**), PI3K inhibitor AS605240 (**B**), AKT inhibitor AS6730 (**C**), and mTOR inhibitor rapamycin (**D**) on nociceptive threshold after 14 days of chronic postoperative pain (CPOP). Dose-response curves on the bottom of each figure (**a′**,**b′**,**c′**,**d′**) were generated by nonlinear regression analysis and represent the inhibition of the nociceptive threshold at hour 3 (in gray) of the von Frey filament test. Log IC_50_ represents the Log of the dose required to inhibit 50% of the nociceptive response. Data are presented as the means ± SEM; n = 5–6 per group; *** *p* < 0.001 compared with naïve group; ## *p* < 0.01 and ### *p* < 0.001 compared with CPOP group, according to two-way RM-ANOVA followed by Bonferroni multiple comparison test. The red arrow indicates the day of administration of each drug. BL: baseline.

All data were presented as the mean and standard error of the mean (mean ± S.E.M.). Nociceptive threshold data (Figure 2) were analyzed by two-way repeated measure analysis of variance (two-way RM ANOVA) followed by Bonferroni’s multiple comparison test when appropriate using treatment and repeated time as factors. For Western blotting data, unpaired Student’s *t*-test was used to compare differences between CPOP and naïve control group, and one-way ANOVA followed by Bonferroni’s multiple comparison test was used to analyze TRPV1 involvement in spinal mTOR activation during CPOP. Results with *p* < 0.05 were considered statistically significant. Furthermore, dose–response curves for the inhibition of the nociceptive threshold at hour 3 of measurement in the von Frey filament test were generated by nonlinear regression analysis to calculate the IC_50_ for each inhibitor. In addition, they compared the findings related to treatment to different doses. All analyses and graphs were created using GraphPad Prism version 8 (GraphPad Software, Inc., San Diego, CA, USA).

## 3. Results

### 3.1. Spinal Involvement of TRPV1 and PI3K/AKT/mTOR Pathway in CPOP-Induced Nociception

After 14 days, a significant reduction in the nociceptive threshold in the CPOP group compared to the naïve group in all experiments was found (Figure 2A–D). Pretreatment with the low dose of TRPV1 antagonist SB-366791 (2.87 µg/5 µL) prevented the nociceptive response from 1 to 3 h, whereas a high dose (28.7 µg/5 µL) induced a more pronounced antinociceptive response (from 1 to 7 h; Figure 2A), with Log IC_50_ at 0.22 (Figure 2a’). In addition, pretreatment with the low dose of PI3K inhibitor AS605240 (0.5 µg/5 µL) prevented the nociceptive response only at hour 3. The high dose of AS605240 (5 µg/5 µL) induced an antinociceptive response from 3 to 7 h (Figure 2B), with Log IC_50_ at 0.10 (Figure 2b’). Concerning the treatment with AKT inhibitor AS6730, the analysis showed that the low dose (0.1 µg/5 µL) induced a more pronounced antinociceptive response (from 1 to 7 h) compared to the high dose (1 µg/5 µL), which induced an antinociceptive response from 1 to 5 h (Figure 2C), with Log IC_50_ at −1.09 (Figure 2c’). Finally, pretreatment with the low dose of mTOR inhibitor rapamycin (0.1 µg/5 µL) prevented the nociceptive response from 1 to 3 h, whereas a high dose (1 µg/5 µL) induced an antinociceptive response from 1 to 5 h (Figure 2D), with Log IC_50_ at −8.87 (Figure 2d’). The statistical data are described in Supplementary Material Table S1.

### 3.2. TRPV1 Protein Levels During CPOP

**Figure 3:**
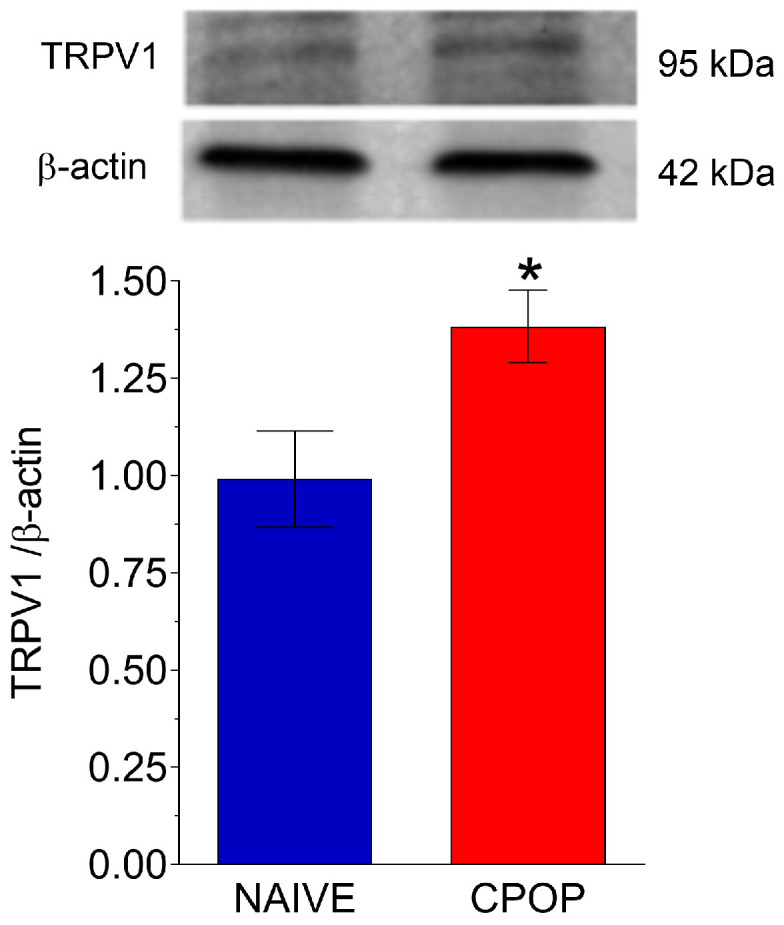
TRPV1 protein levels in the spinal cord 14 days following the surgery. Data are presented as mean ± S.E.M.; n = 4 per group; * *p* < 0.05 compared with the naïve control group (Student’s *t*-test for independent samples).

In addition to the behavioral experiments, Western blot analysis showed an increase in TRPV1 levels in the CPOP group as compared to the naive group (Figure 3).

### 3.3. PI3K/AKT/mTOR Protein Levels During CPOP

**Figure 4:**
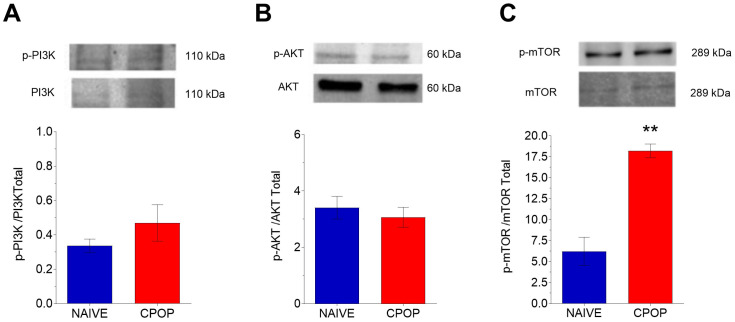
Effect of chronic postoperative pain (CPOP) on PI3K (**A**), AKT (**B**), and mTOR (**C**) protein levels in the spinal cord measured 14 days after the surgical procedure. Data are presented as the mean ± S.E.M.; n = 4 per group; ** *p* < 0.01 compared with the naïve group (Student’s *t*-test for independent samples).

When evaluating the PI3K/AKT/mTOR protein levels, no change was found in the spinal expression of PI3K protein levels (Figure 4A) and AKT after 14 days of CPOP (Figure 4B). However, the CPOP group presented a significant increase in the mTOR protein level expression compared with the naïve group (Figure 4C).

### 3.4. TRPV1 Involvement in mTOR Protein Activation in the Spinal Tissue

**Figure 5:**
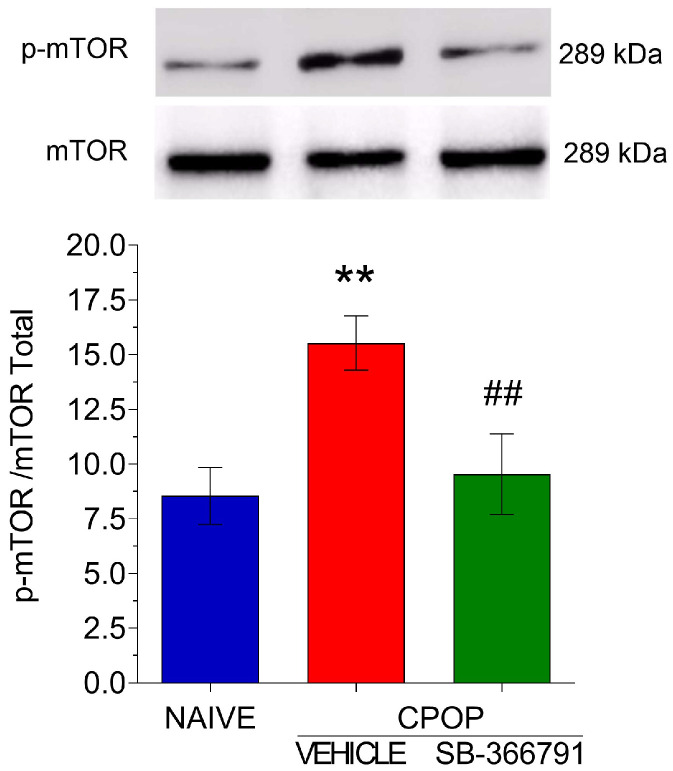
Involvement of TRPV1 in the spinal mTOR activation in spinal tissue. Data are presented as the mean ± SEM; n = 3 per group. ** *p* < 0.01 compared with naïve control group; ## *p* < 0.01 compared with CPOP group (one-way ANOVA followed by Bonferroni’s multiple comparison test).

As shown in the previous experiment, after CPOP, an increase in the spinal mTOR protein levels was found. Then, an experiment was conducted to evaluate whether this activation influences mTOR expression. Interestingly, pretreatment with the TRPV1 antagonist SB-366791 at a dose of 28.7 µg/5 µL prevented the increase in mTOR expression, suggesting that TRPV1 may influence the activation of this protein (Figure 5).

## 4. Discussion

The present study showed that TRPV1 and the PI3K/AKT/mTOR pathway may be involved in CPOP pathogenesis and that TRPV1 is involved with mTOR activation.

Previous studies have demonstrated the involvement of the PI3K/AKT/mTOR pathway in different pain models, such as neuropathic, oncological, and inflammatory [8,10,11,21,22,23]. Although this pathway is involved in the nociception process, there is a gap in the literature in demonstrating how this pathway is activated. Thus, we hypothesized that TRPV1 could be activated during CPOP, activating the PI3K/AKT/mTOR pathway.

At a peripheral level, during tissue injury, several mediators are released, such as nerve growth factor, prostaglandin E2, and adenosine 5′-triphosphate. These effectors can bind to specific TRPV1 sites, with the consequent activation of intracellular proteins, which can alter the opening of the ion channels responsible for reducing the activation threshold of nociceptive neurons [21]. The increased TRPV1 expression in peripheral neurons verified this process. Furthermore, it was found that in TRPV1 knockout mice, there was no change in the nociceptive threshold in different pain models and no change in its expression [24,25,26].

At the spinal level, studies have demonstrated an important role for TRPV1 as a presynaptic modulator during the nociceptive process, mainly facilitating the release of the cytokine TNF-α and the excitatory neurotransmitter glutamate [27,28]. In addition, a study demonstrated that the intrathecal administration of an NMDA glutamate receptor antagonist reversed the hyperalgesia to thermal in mice with postoperative pain induced by paw skin incision [24]. Studies have also suggested an important role for TRPV1 expressed in postsynaptic spinal neurons during nociception. TRPV1 activation increases the frequency of spontaneous inhibitory postsynaptic currents in the dorsal horn in GABAergic or glycinergic neurons, reducing their inhibitory functions [29,30].

The intracellular signaling cascade involved in TRPV1 effects, especially in nociception, is poorly understood. A previous study evaluated the effect of TRPV1 activation by prostaglandin E2, discovering the involvement of the extracellular signal-regulated kinase (ERK) pathway [31]. Additional evidence has also demonstrated that the intracellular PI3K/AKT/mTOR pathway may be involved in the activation and biological effects of TRPV1 [11,21].

The PI3K/Akt/mTOR pathway is involved in the cell cycle, proliferation, cancer, and in some types of pain, mainly neuropathic pain [8,9,10,11,21,22,23,32]. PI3K activation converts phosphatidylinositol 4,5-bisphosphate to phosphatidylinositol 3,4,5-trisphosphate (PIP3), which activates phosphoinositide-dependent kinase-1, resulting in AKT and mTOR activation [8,9,10,11,32].

In addition, mTOR activation can lead to the production of mediators involved in the nociceptive synapse, such as proinflammatory cytokines [33,34,35], which are involved in central pain sensitization [36]. Although the role of the PI3K/AKT/mTOR pathway has been well demonstrated in pain pathophysiology, no study has investigated its involvement in CPOP.

Regarding the site that both TRPV1 and mTOR are involved in during postoperative pain, studies demonstrated that TRPV1 is mainly present in sensory neurons and DRG neurons [37,38,39,40], while mTOR was found in peripheral neurons, DRG neurons, and spinal microglia [10,21,41]. However, these studies used a model of postoperative pain induced by skin incision in which recovery and healing are faster than the model used in the present study.

One of the first studies investigating AKT activation via TRPV1 demonstrated that the administration of TRPV1 agonists promoted AKT phosphorylation and endothelial nitric oxide synthase (eNOS) upregulation in endothelial cells in vitro [12]. In addition, TRPV1 activation increased Akt phosphorylation and eNOS levels in aortas from wild-type mice but failed to activate eNOS in TRPV1-deficient aortas. Around the same time as this study, another study found TRPV1 participation in cold stress-induced brain injury in mice through PI3K/AKT/mTOR pathway activation [22]. Another important study demonstrated that TRPV1 and PI3K activation was related to inflammatory cytokine hypersecretion in bronchial epithelial cells (HBE16) stimulated with the TRPV1 agonist capsaicin [42].

Thus, this previous evidence supports our hypothesis that TRPV1 and PI3K/AKT/mTOR pathway inhibition attenuate CPOP-induced nociception. Although pharmacological results demonstrated a possible spinal involvement of the pathway, our protein expression results indicated only the spinal involvement of TRPV1 and mTOR in CPOP. In addition, a TRPV1 blockade was found to reduce mTOR protein expression. Supporting this finding, Gunthorpe et al. [43] demonstrated that the TRPV1 antagonist, which was used in the present study, is a potent and exclusive blocker for these channels. These authors performed a series of FLIPR experiment binding assays and functional screens and demonstrated that SB-366791 produces little or no effect on a wide range of enzymes, ion channels, GPCRs, and other proteins. This indicates that the effect found by this antagonist in the present study is directly due to the blockade of TRPV1 and not of mTOR.

Given these results, we suggest that the mTOR activation by TRPV1 found in our study may have occurred through other intracellular signaling pathways, such as mitogen-activated protein kinase (MAPK), ribosomal protein s6 kinase beta-1 (s6k1), and tank-binding kinase 1 (TBK1) [44]. Furthermore, the protein complex around mTOR, such as Raptor and mLST8, when activated by MAPKs ERK1/2, JNK, or PKA can also promote mTOR activation [31,44].

Another explanation for our results would be the binding site evaluated in some proteins of the pathway. In the present study, according to the antibodies used, the following phosphorylation sites were evaluated: p110γ for PI3K, Thr 308 for AKT, and Ser 2481 for mTOR. Although PI3K p110γ and p-AKT1 Thr 308 levels were not upregulated in CPOP, other phosphorylation sites may be involved, since previous studies demonstrated that spinal mTOR activation is PI3K- and AKT-dependent during pain [8,45]. Therefore, further studies are needed to investigate the main pathways involved in mTOR activation through TRPV1 during CPOP.

The present study has some limitations, such as the fact that the experiments were performed only on male animals. Although studies have shown that the highest incidence of chronic postoperative pain is in females [46], males also present pain, and in the present study, it was not possible to make this comparison due to the lack of availability of animals. In addition, a future study is under development in our group to co-localize TRPV1 and mTOR in glial cells and neurons in the spinal cord during CPOP, which was impossible to verify in the present study. Other limitations were the need to euthanize the animals on different days after CCI to evaluate protein expression, thus correlating with behavioral results, and the lack of a naïve group pretreated with the TRPV1 antagonist SB-366791 to confirm that the effect of this substance is not on mTOR. However, it has already been previously demonstrated that SB-377791 is highly selective for TRPV1 [47].

## 5. Conclusions

The present study suggests that TRPV1, as well as the PI3K/AKT/mTOR pathway, are involved in CPOP pathogenesis at the spinal level. In addition, our findings provide evidence that TRPV1 may activate mTOR during this process. Furthermore, the present study proposes that both TRPV1 and mTOR may be two important targets for the development of pharmacological and nonpharmacological therapies for the control of CPOP. Therefore, studies should be conducted to further elucidate this pathway and contribute to the development of more effective treatment strategies for CPOP.

In addition, many clinical trials are under development, intending to inhibit the action of TRPV1 and consequently, control pain, especially of inflammatory and neuropathic origins. However, the great challenge for the pharmaceutical industry is the development of TRPV1 inhibitors that produce fewer adverse effects, such as hyperthermia and the alteration of the noxious heat sensation, which have been frequently found in clinical trials [48]. In addition, another strategy for controlling chronic pain would be the inhibition of mTOR, since some preclinical trials have demonstrated this effect [49]. Clinical studies investigating cancer control have also shown that mTOR inhibition may be promising and cause few adverse effects [50]. Therefore, given the data found in the present study, future clinical trials involving the development of mTOR inhibitors will be important as a new strategy for controlling chronic pain, especially pain originating after surgery.

## Data Availability

Data are contained within the articles.

## Supplementary Materials

The following supporting information can be downloaded at: https://www.mdpi.com/article/10.3390/brainsci15010053/s1, Table S1: Statistical analysis.
