# Supplementary material for: Spinal Involvement of TRPV1 and PI3K/AKT/mTOR Pathway During Chronic Postoperative Pain in Mice
Source: Brain Sci. 2025 Jan 8;15(1):53. doi: 10.3390/brainsci15010053 (PMC11763465; doi:10.3390/brainsci15010053)
Supplement: Supplementary file 1 [file brainsci-15-00053-s001.zip › brainsci-3333218-supplementary.pdf]

Supplementary material Table S1 - table containing statistical analyses.

**Figure 2**	**Statistic**	
	2-way RM ANOVA	
**Figure 2A** – Effect of TRPV1 antagonist SB-366791 on CPOP-induced nociception	effect of the treatment	$F_{(4,24)}$=31.66; p<0.001
	effect of the time	$F_{(6,144)}$= 32.90; p<0.001
	treatment x time interaction	$F_{(24,144)}$=8.140; p<0.001
**Figure 2B** – Effect of PI3K inhibitor AS605240 on CPOP-induced nociception	effect of the treatment	$F_{(4,24)}$=82.05; p<0.001
	effect of the time	$F_{(6,144)}$= 49.43; p<0.001
	treatment x time interaction	$F_{(24,144)}$=8.422; p<0.001
**Figure 2C** – Effect of AKT inhibitor AS6730 on CPOP-induced nociception	effect of the treatment	$F_{(4,24)}$=199.8; p<0.001
	effect of the time	$F_{(6,144)}$= 21.62; p<0.001
	treatment x time interaction	$F_{(24,144)}$=8.711; p<0.001
**Figure 2D** – Effect of rapamycin on CPOP-induced nociception	effect of the treatment	$F_{(4,24)}$=54.48; p<0.001
	effect of the time	$F_{(6,144)}$= 20.95; p<0.001
	treatment x time interaction	$F_{(24,144)}$=7.805; p<0.001

**Figure 3**	**Statistic**	
	Student's t test for independent samples	
**Figure 3** – Spinal expression of TRPV1	*t*-test	t_6 = 2.532, p<0.05

**Figure 4**	**Statistic**	
	Student's t test for independent samples	
**Figure 4A** – Spinal expression of PI3K	*t*-test	T_6 = 1.176, p>0.05
**Figure 4B** – Spinal expression of AKT	*t*-test	t_6 = 0.633, p>0.05
**Figure 4C** – Spinal expression of mTOR	*t*-test	t_5 = 5.687, p<0.01

**Figure 5**	**Statistic**	
	One-way ANOVA	
**Figure 5** – Effect of TRPV1 antagonist SB-366791 on spinal mTOR activation during CPOP	effect of the treatment	$F_{(2,6)}$=19.44; p<0.01
